# Retinoic acid has the potential to suppress endometriosis development

**DOI:** 10.1186/s13048-015-0179-6

**Published:** 2015-07-31

**Authors:** Yoshiaki Yamagata, Eiichi Takaki, Masahiro Shinagawa, Maki Okada, Kosuke Jozaki, Lifa Lee, Shun Sato, Ryo Maekawa, Toshiaki Taketani, Hiromi Asada, Hiroshi Tamura, Akira Nakai, Norihiro Sugino

**Affiliations:** Department of Obstetrics and Gynecology, Yamaguchi University Graduate School of Medicine, Minamikogushi 1-1-1, Ube, 755-8505 Japan; Department of Biochemistry and Molecular Biology, Yamaguchi University Graduate School of Medicine, Minamikogushi 1-1-1, Ube, 755-8505 Japan

## Abstract

**Background:**

Despite endometriosis is common estrogen dependent disease afflicting women in reproductive age, the pathogenesis has not been fully elucidated. Retinoic acid has various functions in cells as biologic modulator, and aberrant retinoid metabolism seems to be involved in the lesions of endometriosis. In order to evaluate the potential of all-trans retinoic acid (ATRA) for therapeutic treatment, a transcriptome analysis and estradiol measurements in cultured endometriotic cells and tissues were conducted.

**Methods:**

The mRNA expression levels in ATRA-treated endometriotic stromal cells (ESC) isolated from ovarian endometrial cysts (OEC) were investigated. Estradiol production in OEC tissues was also investigated.

**Results:**

In the isolated ESC culture supplemented with ATRA for four days, total RNA was extracted followed by a transcriptome analysis using GeneChip. Forty-nine genes were upregulated and four genes were down-regulated by the ATRA treatment. Many upregulated genes were associated with the negative regulation of cellular proliferation. In addition, ATRA treatment decreased the mRNA expression of 17-beta-dehydrogenase 2 (*HSD17B2*) which converts estradiol into estrone in a dose-dependent manner, and the ELISA measurements indicated that estradiol production in the OEC tissue was inhibited by ATRA treatment.

**Conclusions:**

Retinoic acid has the potential to suppress endometriosis development.

## Introduction

Endometriosis is a common gynecological disease affecting approximately 10 % of reproductive age females. This condition is characterized by the ectopic localization of endometrial-like tissue in the pelvic cavity. As a result of the development of the disease, chronic inflammation is induced in the pelvic cavity, and symptoms such as chronic pelvic pain and infertility subsequently affect the patient’s health. Although numerous studies have been conducted to elucidate the pathogenesis including the origin, loss of control of cell proliferation and local steroidogenesis, etc., no clear answers have been obtained thus far.

Vitamin A has diverse essential physiological roles in embryonic development, reproduction, vision, immune cell development, and various nervous functions [1–8]. Vitamin A performs these roles through its derivatives called retinoids. After retinol acid is brought into cells by retinol binding protein, which is the principle and specific carrier of retinol in the blood, and is subsequently combined with retinoic acid receptors that form heterodimers with the retinoid X receptor at specific promoters and modulate transcription, it functions as a biologic modulator affecting immunomodulatory, anti-inflammatory and cell developmental activities, etc. All-*trans* retinoic acid (ATRA) is the active form of the metabolite of vitamin A and produced from the metabolic conversion of retinol.

The uterine endometrium is a retinoic acid accumulated tissue, and has been recognized as being necessary for normal endometrial cell differentiation and functions [9–12]. Recent studies suggest the possibility that aberrant retinoid metabolism is involved in the pathophysiology of endometriosis [13–18]. Our previous study demonstrated many aberrant DNA methylation lesions accompanying an abnormal mRNA expression in isolated endometriotic stromal cells derived from ovarian endometrial cysts (choESC) [19]. Of these genes, the *STRA6* and *HSD17B2* genes show an abnormally low expression and high level of DNA methylation in cases of ovarian endometriosis. *STRA6* is an essential cell surface receptor for retinol binding protein and is necessary for the uptake of retinol into cells. *HSD17B2* converts estradiol into estrone. Therefore, a low expression of *STRA6* and *HSD17B2* results in the enhanced endogenous synthesis of estradiol. An elevated estradiol concentration within the endometriotic tissue promotes the development of endometriosis. Moreover, reduced ATRA levels are observed in clinical endometriotic lesions compared to the eutopic endometrium [18], and ATRA has inhibitory effects on mouse endometriotic implants in an in vivo endometriosis model [20]. Accumulating evidence showns that an aberrant retinoic acid metabolism is a critical factor for the development of endometriosis.

In this study, we examined the effects of ATRA on the gene expression in isolated and cultured choESC. We also evaluated the effect of ATRA on estradiol production, the key modulator of endometriosis development.

## Material and methods

The study protocol was reviewed and approved by the Institutional Review Board of Yamaguchi University Graduate School of Medicine. Informed consent was obtained from the participants before the collection of any samples. All experiments involving the handling of human cells and tissues were performed in accordance with the tenets of the Declaration of Helsinki.

### ESC isolation, culture and total RNA isolation

Ovarian endometrial cysts (OEC) were obtained from three subjects (aged 24 – 39 years) during the proliferative phase. None of the subjects used any hormonal therapy for at least 3 months prior to operation. ESC was isolated as previously reported [19]. Briefly, OCE were washed with phenol red-free Dulbecco’s modified Eagle’s medium (DMEM) (Invitrogen, Paisley, UK) containing Glutamax (Invitrogen), 50 mg/ml of streptomycin (Invitrogen) and 50 IU/ml of penicillin (Invitrogen) and then minced into small pieces measuring <1 mm^3^. Then, enzymatic digestion of the minced tissues with 0.2 % collagenase (Sigma, St. Louis, MO, USA) was performed in a shaking incubator for two hours at 37 °C, after which the endometrial stromal cells were separated using filtration through a 70 mm nylon mesh. The filtrates were washed three times. The choESC were seeded in 75 cm^2^ tissue culture flasks and grown until confluence in phenol red-free DMEM containing Glutamax, antibiotics and 10 % dextran-coated charcoal-stripped fetal calf serum (Biological Industries, Kibbutz Beit Haemek, Israel) at 37 °C in 95 % air and 5 % CO2. The homogeneity of the isolated ESC preparation was 98 %, which was verified by immunocytochemistry using an antibody against vimentin, a specific marker of stromal cells. If necessary, the cells were subcultured in another 75 cm^2^ tissue culture flask.

In order to investigate transcriptional changes after ATRA treatment in the cultured choESC, ATRA was added to the media at a final concentration of 10^−7^ M. Separately, 10^−9^, 10^−8^ and 10^−7^ M of ATRA was added to the media for the *HSD17B2*-mRNA expression analysis. DMSO was added to the media as a vehicle control. The medium was changed every other day, and after four days of incubation, the cells were harvested and frozen at−80 °C until RNA extraction.

Total RNA was extracted using an RNeasy kit from QIAGEN (Valencia, CA, USA) in accordance with the manufacturer’s instructions.

### Transcriptome analysis

In order to evaluate the integrity of the RNA, a microfluidic analysis was performed using an Agilent 2100 Bioanalyzer with the RNA6000 nano kit (Agilent Technologies, Palo Alto, CA, USA). For the microarray analysis, we used only RNA samples whose RNA integrity number (RIN) was greater than 8.5. The gene expression was analyzed using a GeneChip® Human Gene 1.0 ST Array (Affymetrix, Santa Clara, CA, USA) containing 764,885 probes (and supporting 28,869 genes). Target cDNA was prepared from 200 ng of total RNA with the Ambion® WT Expression kit (Ambion, Austin, TX, USA) and the Affymetrix® GeneChip® WT Terminal Labeling kit (Affymetrix). Hybridization to the microarrays, washing, staining and scanning were performed using the GeneChip® system (Affymetrix) composed of a Scanner 3000 7G Workstation Fluidics 450 and a Hybridization Oven 645. The scanned image data were processed using the Affymetrix® Expression Console™ Version 1.1. The fold-change for each gene was evaluated using a Gene Expression Analysis with the Partek® Genomics Suite 6.5 software program (Partech, Münster, Germany). Genes with an expression level greater than 2-fold or less than 0.5 were recognized as being significantly different.

### Quantitative RT-PCR

Among the genes differentially expressed in the ATRA-treated choESC compared to the control cells, we focused on the *IRF8, TNFSF13B, WNT4, RARRES1, IGFBP3, PSMB9, RARRES3, IGFBP6, CYP26B1, IDO1* and *RARE* genes associated with negative cellular proliferation. In order to validate the results of the transcriptome analysis, real-time RT-PCR was conducted on these genes using the same samples. The *HSD17B2* mRNA expression was also examined.

The RT reactions were performed with the PrimeScript RT Master Mix (TAKARA, Ohtsu, Japan) according to the manufacturer’s protocol. Briefly, 0.5 μg of total RNA was incubated with 4 μl of 5x PrimeScript RT Master Mix in 20 μl of reaction mixture at 37 °C for 15 min, and reverse transcriptase was inactivated by heating the samples at 85 °C for 5 s. The complementary DNA (cDNA) was immediately used for PCR. All PCR reactions were performed using SYBR Premix Ex Taq (TAKARA) and a LightCycler instrument (Roche Applied Science, Basel, Switzerland). Briefly, 2 μl aliquots containing cDNA were amplified in a total volume of 20 μl containing 4 μl of 5x SYBR PreMix Ex Taq and 0.2 μM of each primer. As an internal control for RT-PCR, TATA box-binding protein (TBP) cDNA was also amplified. The specific primer sets, except for TBP, were designed using the Primer3 software program (frodo.wi.mit.edu), while primers for TBP were synthesized according to a previous report [21]. The primer sequences are described in Table 1. Shuttle PCR was performed in 40 cycles as follows: pre-incubation for 10 s at 95 °C, denaturation for 5 s at 95 °C and annealing/extension for 20 s at 60 °C. All samples were run in duplicate. The melting curves of the products were obtained after cycling with a stepwise increase in temperature from 55 to 95 °C. At the end of the 40 cycles, the reaction products were separated electrophoretically on an agarose gel and stained with ethidium bromide for visual confirmation of the PCR products.

**Table 1:** Primer sequences used for quantitative RT-PCR

Gene ID	Forward primer	Reverse primer	Product size (bp)
*TNFSF13B*	GGAGAAGGCAACTCCAGTCA	GCAATCAGTTGCAAGCAGTC	92
*IRF8*	GTCTTCGACACCAGCCAGTT	GGCCATATCCGGAAACTCTT	114
*RARRES3*	GTGAGCAGGAACTGTGAGCA	CAAAAGAGCATCCAGCAACA	136
*RARRES1*	ACGGCTCATCGAGAAAAAGA	GAAAGCCAAATCCCAGATGA	151
*IGFBP3*	GGGGTGTACACATTCCCAAC	AGGCTGCCCATACTTATCCA	116
*IGFBP6*	GAATCCAGGCACCTCTACCA	GGTAGAAGCCTCGATGGTCA	173
*CYP26B1*	ACACGGTGTCCAATTCCATT	GCCTCCTGGTACACGTTGAT	172
*IDO1*	GGCAAAGGTCATGGAGATGT	TCCAGTTTGCCAAGACACAG	127
*PSMB9*	ACCAACCGGGGACTTACC	GTCAAACTCCACTGCCATGA	70
*RARB*	GAAACAGGCCTTCTCAGTGC	TTGCTGGGTCGTCTTTTTCT	137
*WNT4*	GCTGTGACAGGACAGTGCAT	GCCTCATTGTTGTGGAGGTT	169
*TBP*	TGCACAGGAGCCAAGAGTGAA	CACATCACAGCTCCCCACCA	132
*HSD17B2*	TGGAACTGTGGAGGTCACAA	CCACTTGGAAAGCTCCAGTC	178

### Tissue culture and estradiol assay

The OEC culture was performed as previously reported with modifications [22]. Briefly, the ovarian endometrial cyst wall was surgically removed from the ovary. The tissue was minced into pieces less than 1.5 mm in maximum diameter, and the minced tissue (100 mg wet weight) was randomly aliquoted to the experimental group. Incubation were performed in triplicate in plastic culture dishes with 1 ml phenol red-free DMEM containing Glutamax (Invitrogen), 50 mg/ml of streptomycin (Invitrogen), 50 IU/ml of penicillin (Invitrogen) and 10 % dextran-coated charcoal-stripped fetal calf serum (Biological Industries) at 37 °C in 95 % air and 5 % CO2. ATRA was added to the media at a final concentration of 10^−7^ M. The tissue was also cultured in control medium containing 0.01 % dimethyl sulfoxide. Two days later, the media were aspirated, centrifuged and frozen at −20 °C until the estradiol assay. The estradiol concentration in the culture medium was measured using a human estradiol ELISA kit (Cusabio Biotech, Wuhan, China).

### Statistical analysis

Comparison between groups were performed by ANOVA followed by post hoc comparisons using Turkey-Kramer honestly significant difference test. Values of *P* < 0.05 was considered to be statistically significant. The statistical analyses were performed using the R version 2.12.0 software program.

## Results

### Transcriptome analysis and quantitative RT-PCR

Of the 28,869 human genes identified in our gene index, 49 genes were upregulated and only four genes were downregulated in the ATRA-treated choESC compared to the control choESC (Table 2).

**Table 2:** Results of the GeneChip microarray. Changes observed in the mRNA levels in the ATRA-treated choESC compared to the control cells

Gene symbol	Fold-change	*P*-value
RARB	7.77	0.0001
RARRES1	7.31	0.0089
DHRS3	6.09	0.0006
LXN	5.30	0.0016
ADH1C	4.21	0.0047
CD22	4.09	0.0210
IRF8	3.97	0.0168
RARRES3	3.83	0.0142
TNFSF10	3.68	0.0169
IDO1	3.51	0.0369
GBP4	3.43	0.0032
ALDH1A1	3.31	0.0260
LGALS9B	3.06	0.0022
ANO3	3.04	0.0092
LGALS9	2.98	0.0101
LGALS9C	2.91	0.0034
GALNT12	2.89	0.0317
IRF1	2.87	0.0007
IGFBP6	2.80	0.0001
SLCO4C1	2.79	0.0021
TRPC4	2.76	0.0121
IGFBP3	2.54	0.0135
MX2	2.45	0.0138
TNFSF13B	2.44	0.0173
CYP26B1	2.30	0.0066
GNG2	2.28	0.0108
LOC100287290	2.28	0.0006
PELO	2.23	0.0459
OAS2	2.20	0.0198
RTP4	2.19	0.0176
IFIT2	2.19	0.0459
C10orf54	2.17	0.0029
CFI	2.14	0.0255
SAMD9L	2.12	0.0342
ACSL5	2.12	0.0462
SAMD9	2.10	0.0025
TMEM140	2.08	0.0162
APOL6	2.08	0.0074
PLK2	2.06	0.0212
CEACAM1	2.06	0.0235
GNG2	2.04	0.0018
PARP14	2.03	0.0113
WNT4	2.02	0.0405
HS6ST1	2.02	0.0207
PSMB9	2.02	0.0122
PTPRJ	2.01	0.0001
ARHGAP20	2.00	0.0210
GRIA1	−2.41	0.0463
POPDC2	−2.56	0.0401
HAS2	−2.61	0.0323
FMO1	−3.32	0.0262

In order to determine the biological relevance of the differentially expressed genes, Gene Ontology and KEGG pathway analyses were performed. Significant related terms were detected as illustrated (Tables 3, 4). The significant highly expressed genes in the ATRA-treated choESC were related to cellular response induced by ATRA stimulation based on the biological process and molecular function ontology analyses. Several terms related to negative regulation of cellular proliferation were also listed. In the KEGG pathway analysis, genes related to retinol metabolism were found to be upregulated (data not shown).

**Table 3:** Gene ontology categories using biological process ontology. The top 21 category terms with a gene count over three and *p* < 0.01 are listed

Term	Count	*P*-value
Response to stimulus	29	0.0035
Immune system process	20	*p* < 0.001
Negative regulation of cellular process	20	*p* < 0.001
Negative regulation of biological process	20	0.0010
Organ development	17	0.0098
Immune response	16	*p* < 0.001
Regulation of cell proliferation	12	0.0016
Defense response	11	*p* < 0.001
Regulation of apoptosis	11	0.0060
Regulation of programmed cell death	11	0.0065
Regulation of cell death	11	0.0066
Negative regulation of cell proliferation	10	*p* < 0.001
Response to other organism	8	*p* < 0.001
Response to biotic stimulus	8	0.0029
Monocarboxylic acid metabolic process	7	0.0037
Blood vessel morphogenesis	6	0.0039
Positive regulation of response to stimulus	6	0.0063
Blood vessel development	6	0.0073
Vasculature development	6	0.0081
Hormone metabolic process	5	0.0019
Regulation of hormone levels	5	0.0067
Positive regulation of protein kinase cascade	5	0.0095
Cellular hormone metabolic process	4	0.0031
Response to lipopolysaccharide	4	0.0066
Secondary metabolic process	4	0.0071
Response to molecule of bacterial origin	4	0.0089

**Table 4:** Gene ontology categories using molecular function ontology. The top seven category terms with a gene count over three gene count and *p* < 0.05 are listed

Term	Count	*P*-value
Catalytic activity	34	0.0426
Signal transducer activity	19	0.0231
Molecular transducer activity	19	0.0231
Carbohydrate binding	7	0.0076
Cytokine activity	6	0.0026
Sugar binding	5	0.0152
Carboxylic acid binding	4	0.0334

**Fig. 1:**
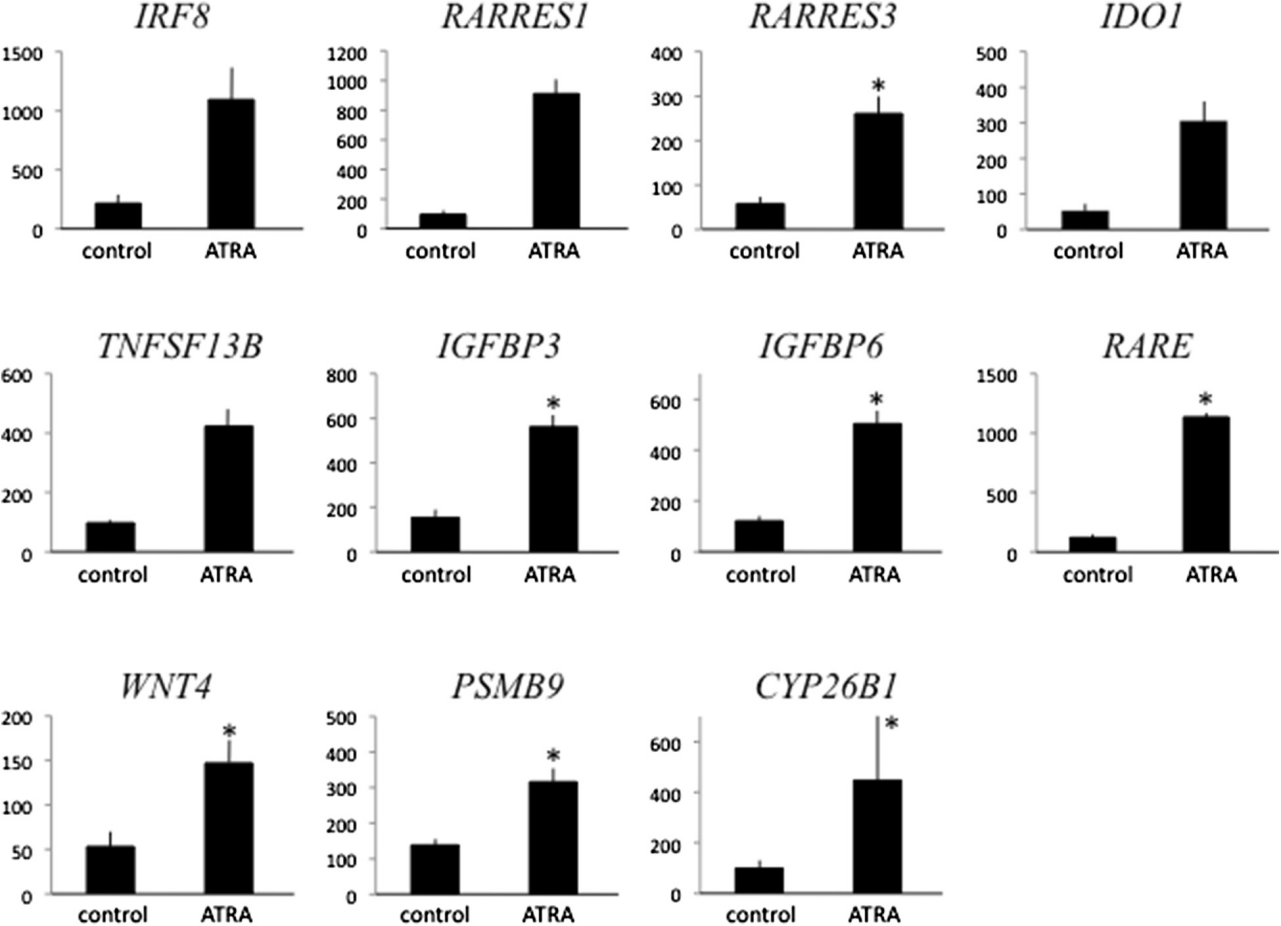
Results of quantitative RT-PCR in the 11 selected genes involved in negative cellular proliferation for validation of the mRNA expression array. The values are shown as mean ± SEM of three experiments. *P < 0.05 vs. control

In order to validate the microarray results, quantitative RT-PCR was performed in 11 selected genes. The mRNAs of all genes were highly expressed following treatment with ATRA, and seven genes showed significant differences (Fig. 1).

### HSD17B2-mRNA expression

**Fig. 2:**
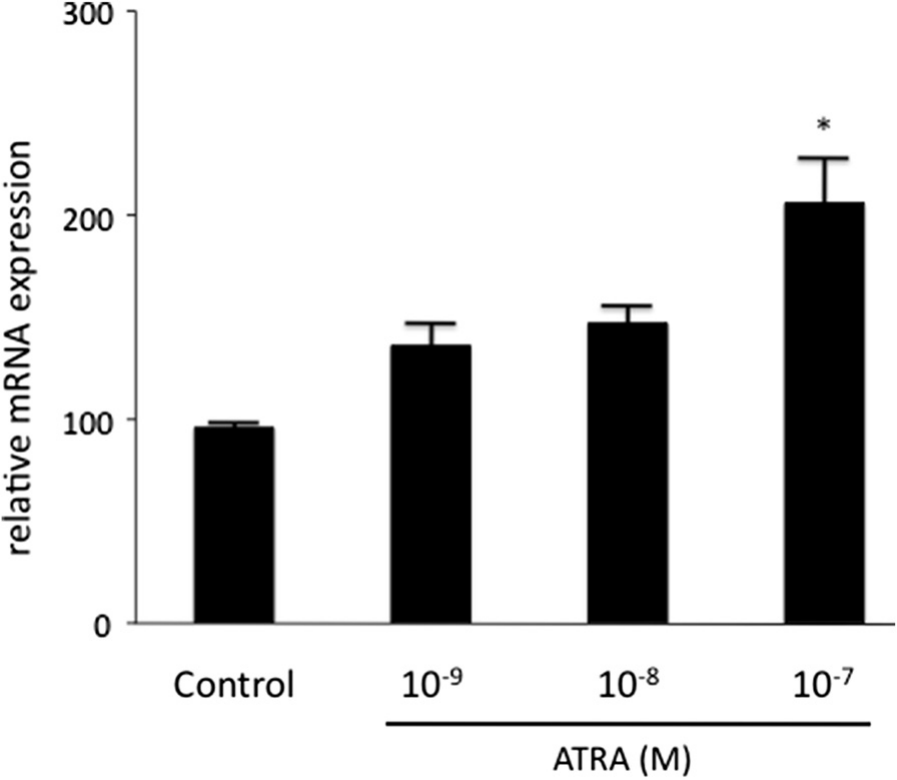
Effect of ATRA on the *HSD17B2*-mRNA expression in the cultured choESC. Cells were obtained from three different individuals, and the cells from a given individual were cultured in triplicate. The cells were treated with the indicated concentrations of ATRA for four days. The values are shown as mean ± SEM of three experiments. *P < 0.05 vs. control, ATRA: 10^−9^ M, and 10^−8^ M

In the choESC culture upon treatment with ATRA, the *HSD17B2*-mRNA expression was increased in a dose-dependent manner. There was a significant difference at a concentration of 10^−7^ M (Fig. 2).

### Estradiol levels in the ATRA-treated endometriotic tissue culture

**Fig. 3:**
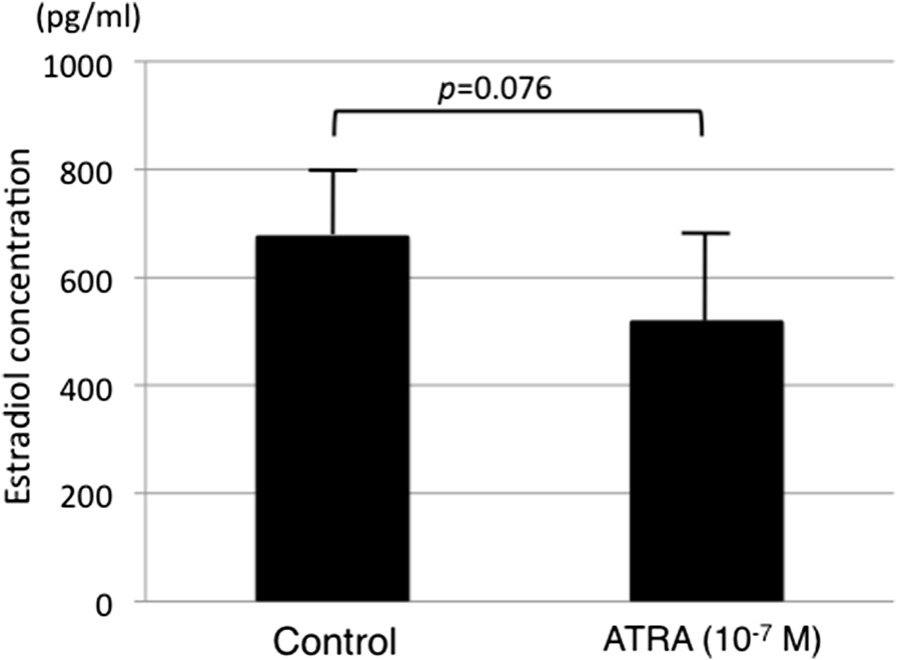
Effect of ATRA on estradiol production in the cultured OEC tissues. Tissues were obtained from four different individuals, and the cells from a given individual were cultured in duplicate. The tissues were treated with 10^−7^ M ATRA for two days. The values are shown as mean ± SEM of four experiments

Endometriotic tissue obtained from OEC was cultured with 10^−7^ M ATRA. The level of estradiol in the medium with ATRA was lower than that observed in the control medium; however, there were no significant differences (Fig. 3).

## Discussion

An association between aberrant retinol metabolism and endometriosis has recently been reported. Pavone M.E. *et al.* demonstrated that progesterone resistance has an influence on retinol uptake and growth-suppressor actions of retinoic acid in endometriotic stromal cells [14]. These authors also illustrated that an abnormal gene expression is involved in retinol uptake and metabolism in the setting of endometriosis, suggesting that an aberrant retinoic acid signaling pathway may affect endometriosis cell survival [15]. Wieser F. *et al.* demonstrated that retinoic acid inhibits the development of endometriotic implants in vivo [20]. In our in vitro study followed by a transcriptome analysis using isolated endometriotic stromal cells, genes upregulated by ATRA treatment were associated with the suppression of cell proliferation. We also conducted a cell proliferation study; however, ATRA had no significant anti-proliferative effect on the cultured choESC for up to four days (data not shown). The fact that the environment in the culture is different from the in vivo conditions and the cultured choESC slowly divide indicates the possibility that the anti-proliferative effects of retinoic acid cannot be detected. Wieser F. *et al.* also reported the same observation suggesting the indirect effects of cell growth inhibition [20]. They postulated the suppressive effect of retinoic acid on IL-6 and MCP-1 production, resulting in peritoneal macrophage differentiation.

Endometriosis is an estrogen-dependent disease. It is well-known that local sex steroid production in endometriotic tissue is upregulated by elevated catalytic enzyme activity, such as that due to aromatase [23–26]. Furthermore, reduced retinoic acid concentrations are observed in endometriotic lesions [18], and we previously reported that the *HSD17B2* expression is suppressed in choESC accompanied by aberrant DNA methylation [19]. Retinoic acid insufficiency results in numerous molecular and functional defects, including *HSD17B2* deficiency, leading to estradiol excess in endometriotic tissue. The present study demonstrated the direct effects of ATRA on the *HSD17B2* expression in choESC. Since ATRA treatment did not alter the DNA methylation status of the *HSD17B2* gene (data not shown), the decreased *HSD17B2* expression is likely due to negative modulation of transcriptional factors. In the transcriptome analysis, the fold change in the expression of the *HSD17B2* gene after treatment with ATRA was +2.22; however, the difference was not significant (*p* = 0.24). This is due to the limitation of our study resulting from the limited quantity of available samples. It is unclear whether the upregulation of genes related to negative cellular proliferation induced by ATRA treatment is due to the direct function of retinoic acid, reduced estradiol production or any other indirect pathways.

In order to investigate the effects of retinoic acid on steroidogenesis, especially estradiol production, an ovarian endometriotic tissue culture was performed. Since the *HSD17B2* expression is much higher in endometrial epithelial cells than in endometrial stromal cells, a tissue culture rather than an endometrial stromal cell culture was conducted in this study. Although the abundance of estradiol slightly decreased with supplementation of ATRA, there were no statistically significant differences. There are some possibilities explaining this result. Retinoic acid has only a weak suppressive effect on the *HSD17B2* gene expression, resulting in the ineffective conversion of estradiol. In the setting of endometriosis, it has not been fully clarified whether retinoic acid upregulates or downregulates sex steroid hormone biosynthesis enzymes. Wickenheisser J.K. *et al.* reported that the gene expressions of *STAR*, *CYP11A* and *CYP17* are stimulated by ATRA in human ovarian theca cells [27]. Steroid hormone synthesis is increased by retinoids in rodents in other organs [28–30]. These observations intimate that it is too premature to draw conclusions in terms of the relationship between retinoic acid and the local estradiol concentrations in patients with endometriosis. Besides sex steroid hormone biosynthesis, it has been reported that retinoic acid decreases estrogen and progesterone receptor-mediated transcriptional activation [31]. In order to elucidate the association between retinoic acid and aberrant hormonal involvement in the pathogenesis of endometriosis, further research is needed.

In summary, the gene expression related to cell proliferation suppression, etc. was upregulated by ATRA treatment in isolated endometriotic stromal cells derived from ovarian endometriotic lesions in the present study. ATRA treatment also had the potential to suppress estradiol production. These results suggest the therapeutic potential of retinoic acid for the treatment of endometriosis.
